# BCSearch: fast structural fragment mining over large collections of protein structures

**DOI:** 10.1093/nar/gkv492

**Published:** 2015-05-14

**Authors:** Frédéric Guyon, François Martz, Marek Vavrusa, Jérôme Bécot, Julien Rey, Pierre Tufféry

**Affiliations:** Molécules Thérapeutiques in Silico, INSERM UMR-S 973, Université Paris Diderot, Sorbone Paris Cité, 75205 Paris Cedex 13, France

## Abstract

Resources to mine the large amount of protein structures available today are necessary to better understand how amino acid variations are compatible with conformation preservation, to assist protein design, engineering and, further, the development of biologic therapeutic compounds. BCSearch is a versatile service to efficiently mine large collections of protein structures. It relies on a new approach based on a Binet–Cauchy kernel that is more discriminative than the widely used root mean square deviation criterion. It has statistics independent of size even for short fragments, and is fast. The systematic mining of large collections of structures such as the complete SCOPe protein structural classification or comprehensive subsets of the Protein Data Bank can be performed in few minutes. Based on this new score, we propose four innovative applications: BCFragSearch and BCMirrorSearch, respectively, search for fragments similar and anti-similar to a query and return information on the diversity of the sequences of the hits. BCLoopSearch identifies candidate fragments of fixed size matching the flanks of a gaped structure. BCSpecificitySearch analyzes a complete protein structure and returns information about sites having few similar fragments. BCSearch is available at http://bioserv.rpbs.univ-paris-diderot.fr/services/BCSearch.

## INTRODUCTION

The large amount of protein structures available, thanks to the efforts of the structural genomics, now constitutes a valuable resource to analyze in depth the impact of amino acid sequence variation on protein conformation. Efficient and large-scale mining of the structures available offers promising perspectives to assist protein engineering and design. It also meets the increasing interest of pharmaceutical industry to develop new biologic entities including large peptides, recombinant proteins, antibodies, immunoconjugates or synthetic vaccines to cite some (1). Indeed, beyond the analysis and the classification of complete protein domains, the focus is progressively moving to a more local level of structure analysis. The present number of entries of the Protein Data Bank (PDB) (2), over 100 000 protein structures, corresponds to several tens of millions of protein fragments of short size (10–20 amino acids) amino acids. There is some challenge to design efficient and fast services to analyze structural similarities with statistical significance.

Whereas numerous approaches have been proposed to classify or align complete protein structures (3,4), fewer methods have been developed for a more local level. Several online facilities have been proposed, which focus on contiguous or linear fragments (5–10). Superimposé (5) combines several search algorithms such as TM-align (11) or CE (12) to search for fragments similar to a query. The FragFinder (6) search engine is based on the comparison of the main chain backbone conformational angles (ϕ and ψ). SA-Mot (7) is based on the encoding of structure as strings of a structural alphabet to search for over-represented conformations among collections of proteins with similar functions. Finally, TopMatch (8) that can generate several alignments between a query and a target protein structure has been recently updated as TopSearch (9). For the comparison of non-sequential motifs, much more complex and slower algorithms have been proposed. These include Rasmot-3D (13), SPRITE and ASSAM (14) or ProSMoS (15), to cite some.

Another important application of structural fragment mining is knowledge-based loop modeling. It implies the search for fragments matching geometric boundary conditions in subsets of the PDB or SCOPe (16). For that purpose, some online services are available at this time. ArchPRED (17) uses secondary structures flanking the missing loop, their relative orientation and the number of missing residues to identify candidate loop conformations. SuperLooper (18) mines the Loop In Protein (LIP) database (19), a comprehensive loop database containing all protein segments up to 15 residues from the PDB, to identify fragments matching geometrical criteria between the two last atoms of the main chain of one flank and the two first of the other. FREAD (20) searches for candidate fragments matching conditions on distances between *C*_α_ of the flanks. The method developed by Peng and Yang (21) does not seem reachable any longer. The recent FALC-Loop (22) uses a *de novo* modeling approach combining fragments for loop generation and thus is not *stricto sensu* based on similarity search.

We have recently introduced a new score based on the Binet–Cauchy kernel, the BC-score. It is a geometric correlation score, with a maximum value that equals 1 indicating perfect similarity, values close to 0 being associated with unrelated conformations and a minimal value of −1 corresponding to mirror conformations. This score addresses two major drawbacks of the widely used root mean square deviation (RMSD). Firstly, it shows better performance in the discrimination of medium-range RMSD values, which leads to the identification of more consistent similarities. Secondly, its statistical significance is independent of fragment size, even for short fragments. Due to the simplicity of the BC-score formulation, BCSearch provides one of the fastest services for large-scale mining of protein structures, being able to undergo several tens of thousands of comparisons per second, which makes possible to mine several thousands of structures per second.

In BCSearch, we take advantage of the speed of computation and the accuracy of the BC-score (23) to propose, in a unified framework, new large-scale mining facilities, some of which previously out of reach. The first application performs a search for similar fragment search within large collections of protein structures, possibly the whole SCOPe, or large subsets of the PDB. Taking advantage of the properties of the BC-score, BCSearch is also able to search for mirror conformations. We have previously illustrated that it is, for instance, able to identify left-handed helices, that, even if rare, are important for the stability of the protein, for ligand binding or as part of the active site (24). Another application called BCLoopSearch is an enhancement of the simple BC-score that makes possible to mine for two disjoint fragments separated by a given number of residues. Finally, it also becomes possible to quantify, for a complete protein structure, the fragments that are specific of the structure, i.e. fragment rarely found into a given collection of structures.

## MATERIALS AND METHODS

### Binet–Cauchy score definition and properties

The Binet–Cauchy score as a measure of conformation similarity has already been described in (23). We only recall here the general concepts.

We only consider the coordinates of the α-carbon atoms of the protein fragments. The coordinates of the *N* residue fragments to be compared are stored in *N* × 3 matrices *X* and *Y*. The coordinate matrices are centered at the origin. We use the structural score derived from the Binet–Cauchy kernel (23). This score, we named Binet–Cauchy score, is the cosine between the Grassmann vectors of *X* and *Y*

(1) \begin{equation*} {\rm BC}\,(X,Y)\ = \ \frac{\det (X^\top Y)}{\sqrt{\det (X^\top X)\,{\det (Y^\top Y)}}}. \end{equation*}

The BC-score is a positive kernel, it is rotation independent and it corresponds to a correlation coefficient between the Grassmann representation of *X* and *Y*, and thus varies from −1 to 1.

Importantly, the BC-score is a flexible score. It is maximal (equals 1) for identical structures. However, it is also possible that BC (*X, Y*) = 1 for two different fragment conformations with RMSD (*X, Y*) > 0. In order to control the admissible amount of flexibility, an additional score, called rigidity score, is used: if we denote *X*_*i*_ (resp. *Y*_*i*_) the coordinates of the *i*th *C*_α_ of the fragment *X* (resp. *Y*) of length *N*. The rigidity score between the two structures is

(2) \begin{equation*} {R^{\prime }(X,Y)}=\max _{1 \le i \le N} {| \Vert X_i\Vert -\Vert Y_i\Vert |} \end{equation*}

(3) \begin{equation*} {R(X,Y)}=\max \lbrace {R^{\prime }(X,Y)}, | \Vert X_{N}-X_1\Vert -\Vert Y_{N}-Y_1\Vert |{\rbrace }. \end{equation*}

It corresponds to a measure of the maximum variation of intra-distances between the residues and the geometric center, and intra-distances between the terminal α-carbons.

The BC-score and the RMSD distance are strongly anti-correlated for very low RMSD values. Both provide comparable measures between close structures. But, since the RMSD averages the distances between atoms, the medium-range and even low-range RMSDs do not imply significative conformation similarity. On the contrary, the BC-score characterizes more precisely global shape similarity. Combined with distortion rate, it allows better discrimination among medium RMSD range hits (23).

Therefore, contrary to the RMSD, the BC-score can be efficiently used to search for fragments in structure databases with a certain amount of flexibility while discarding spurious fragments which cannot be structurally aligned with the query.

## IMPLEMENTATION

### Data sets

BCSearch can mine large collections of protein structures. Presently, two collections of structure have been considered. The first corresponds to the complete collections of structural domains of SCOPe version 2.04 (over 190 000 domains in total) (16), for which it is possible to specify any level of the hierarchy using the class.fold.superfamilly.familly scheme—e.g. g.3 for Toxic hairpinKnottins, g.3.3 for cyclotides. In order to make possible analyses on a subset of structures at high resolution only, a second collection denoted as PDB corresponds to a subset of the PDB corresponding to structures resolved using X-ray diffraction, at resolutions better than 1.6, 1.8, 2.0, 2.2 or 2.5 Å, and with an R-value less than 0.25 or 1.0, as defined by the pisces server (25).

### BCSearch services

**Figure 1.**
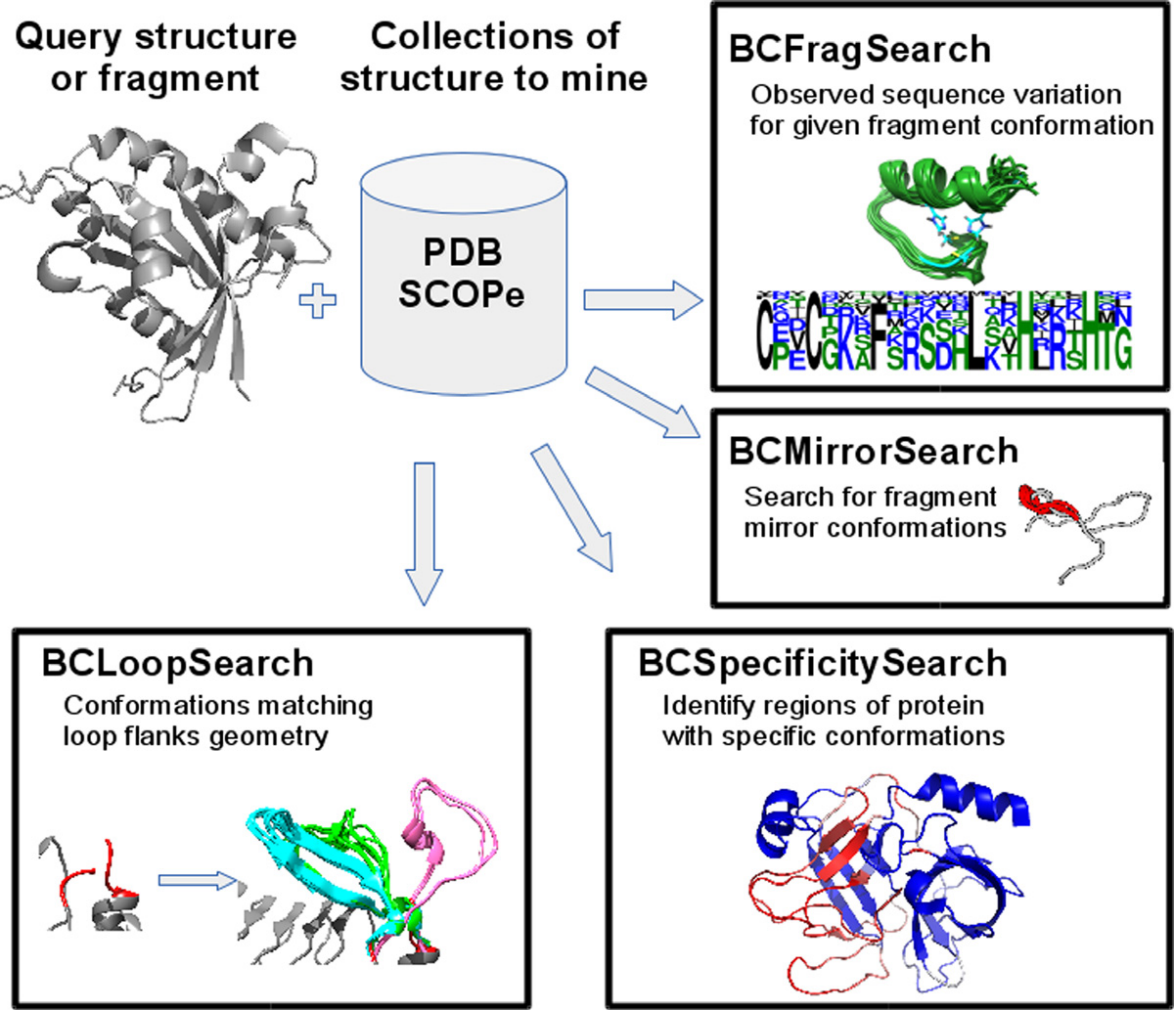
BCSearch services.

Based on the BC-score, BCSearch comes as a collection of services that address different questions, as illustrated in Figure 1.

#### BCFragSearch

The BCFragSearch corresponds to the exhaustive search in a collection of structures of fragments similar to a query. Its aims is to return information about amino acid sequences observed in similar fragments.

#### BCMirrorSearch

The BCMirrorSearch is similar to BCFragSearch except that it corresponds to the exhaustive search in a collection of structures of fragments having conformations anti-similar to a query. Its aim is to return information about the existence of mirror conformations and their amino acid sequences.

#### BCLoopSearch

The BCLoopSearch corresponds to BCFragSearch applied to only the flanking regions of a fragment of interest. It can thus be considered as the search for conformations for fragments of unknown conformation but for which the flanks are known, similarly to the problem of loop modeling. BCLoopSearch uses flanks of four residues. Note that the search is only performed on a geometrical basis, and no control over sequence identity is used during the search. In order to avoid the return of fragments clashing the template structure, a rough checking of steric clashes is performed. Candidates for which at least one inter α-carbon distance to a residue distant of at least three positions in the amino acid sequence is less than a cutoff of 3 Å are discarded.

#### BCSpecificitySearch

This service analyzes a complete protein structure and returns its ‘specific’ parts, i.e. the regions associated with fragment conformations rarely found into a collection of reference structures. This collection can be defined as any SCOPe subset corresponding to a valid class/superfamily/family/fold subset. The search bank can also be defined as the complete SCOPe, excluding some specified SCOP subset. Hence, this service gives the possibility to retrieve fragments specific of a protein structure at a given level of structural similarity. It also permits to search for fragments common to a given SCOP level and specific to this level, that is which are not present at other levels.

We evaluate the fragment specificity with the following score, we denote it as specificity score in the following:

\begin{equation*} sp=1-\frac{N_{{\rm hits}}}{N_{{\rm total}}}, \end{equation*}

where *N*_hits_ corresponds to the number of proteins where a similar fragment is found and *N*_total_ is the total number of proteins in the search bank.

#### Execution times

Typical run times against the full unfiltered SCOPe compendium (over 190 000 domains and 20 millions elementary comparisons) are below 1 min for BCFragSearch, BCMirrorSearch and BCLoopSearch, and on the order of few seconds to 5 min for BCSpecificitySearch, depending on the size of the query and the collection of structures mined.

### Input

As input, BCSearch requires a structure, and in some cases some sequence information. Structures can be uploaded as PDB-formatted files or searched in repositories given a PDB or an SCOPe identifier.

For fragment search and mirror search, a sequence specifying the part of the query structure to use can be input.

For loop search, the complete sequence must be provided. Missing parts of the protein are automatically detected by comparing the sequence to that of the gaped structure.

The collection of structures to mine can correspond to subsets of either the SCOP databank or the PDB at different resolutions. The PDB and SCOP collections can be filtered depending on sequence identity—90, 70, 50 or 30%. Cutoff values can also be set for the BC-score and the rigidity score.

### Output

Results page of the BCSearch services but BCSpecificitySearch return all the hits in a csv file and an interactive table ordered by BCscore, truncated to the best 1000 hits to preserve interactivity. For each match, the data reported are: the name of the query, the name of the hit (PDB or SCOP ID), starting and ending residues number of the query and of the match, BCscore, rigidity value, *P*-value, RMSD and the sequence of the match. The *P*-value and the RMSD are calculated from the query and any hit for BCFragSearch and BCMirrorSearch, and between the flanking regions of the query and the match for BCLoopSearch. When relevant, a sequence logo is also provided. It depicts the sequence variability among the hits. A visualization panel is available thanks to the PV—JavaScript Protein Viewer (http://biasmv.github.io/pv/). For BCSpecificitySearch, a dynamic color gradient allows one to interactively explore the structure at various specificity score values.

## APPLICATIONS

### Fragment mining

**Figure 2.**
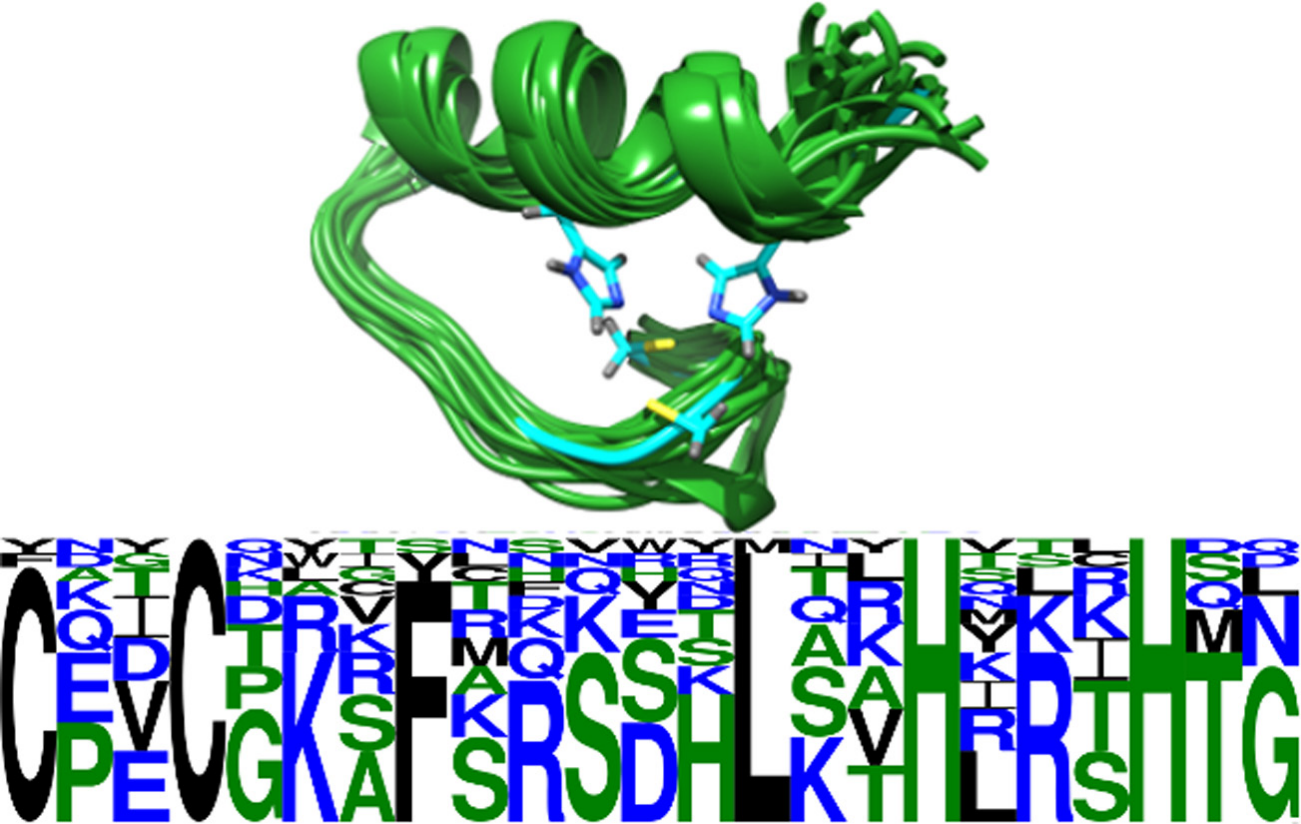
Top: The query structure is the fragment from Cys15 to Gly37 from the human Zinc finger protein (cyan) (PDB: 2EMJ). Twenty eight similar fragments identified by BCFragSearch are depicted in green. Bottom: The corresponding sequence logo shows a conserved C2H2 motif involved in the binding of the zinc.

Figure 2 illustrates a BCFragSearch run applied to the search for fragments similar to the fragment Cys15–Gly37 from the human zinc finger protein (PDB: 2EMJ) that belongs to the superfamily of the beta–beta–alpha-zinc fingers (SCOPe g.37.1). The search was performed against the SCOPe collection at 100% sequence identity, using the default search values of 0.95 and 1. for the BC-score and rigidity, respectively. Twenty eight hits were identified over the 280 proteins of the superfamily. The logo representation of the corresponding sequences clearly shows the C2H2 motif specific of the zinc binding motif. Importantly, similar fragments in the remaining members of the superfamily do have indels in the fragment, highlighting the stringency of the BC-score, which detects such events.

### Candidate loop search

**Figure 3.**
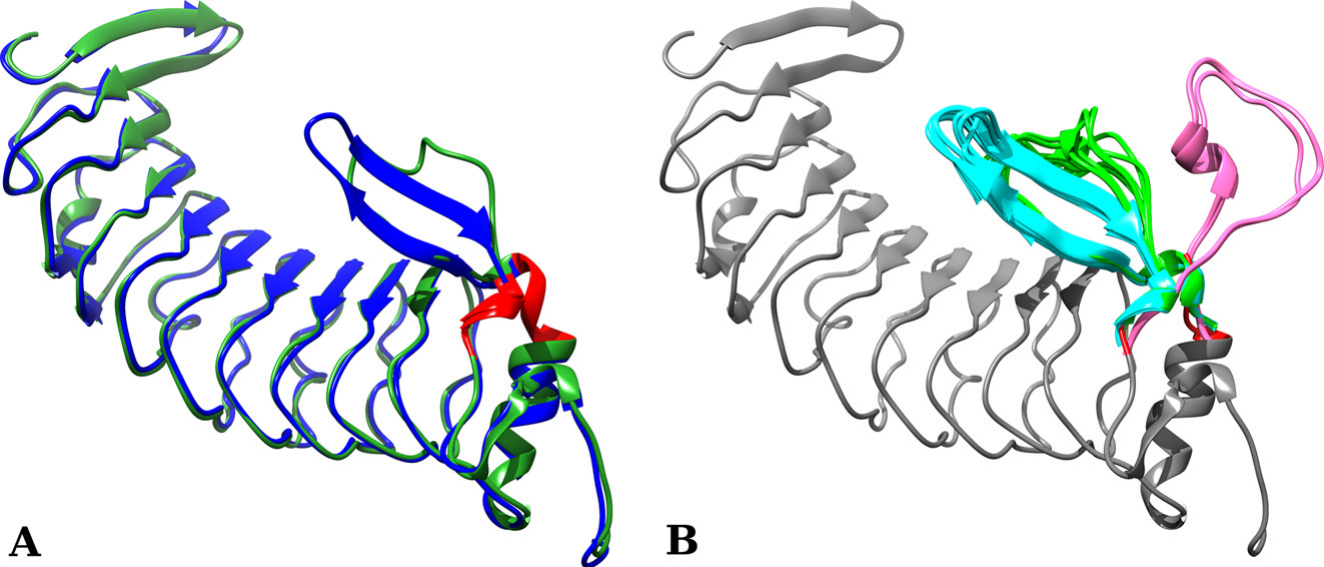
(**A**) The unbound structure of the von Willebrand factor binding domain of glycoprotein Ib alpha is depicted in green (PDB: 1M0Z), and is superimposed onto its bound conformation, taken from the complex with the von Willebrand factor, depicted in blue (PDB: 1M10). The red regions correspond to the four residues flanking fragments used by BCLoopSearch. The 226-242 loop (loop between the two red flanks) undergoes a conformational change of 5.05 Å. The conformation of this region fluctuates from an un-organized segment to an antiparallel beta sheet. (**B**) All matches found by BCLoopSearch superimposed on the query. These matches have been manually clustered: in cyan the one that is close to the bound structure, in light green close to the unbound structure and in pink the outlayer.

To illustrate the BCLoopSearch service, we start from the known complex between the glycoprotein Ib alpha and the von Willebrand factor (PDB: 1M10). Residues from the positions 226 to 242 of the unbound von Willebrand factor binding domain of glycoprotein Ib alpha (PDB: 1M0Z) undergo a large conformational change of 5.05 Å upon binding to the von Willebrand factor (see Figure 3A). Starting from the unbound conformation, we removed residues 226–242 of the moving loop. We then performed a BCLoopSearch against the complete SCOPe collection - 100% sequence identity, using BC-score and rigidity cutoff values of 0.95 and 1.0, respectively. We obtained 20 different conformations. Not only the bound conformation but also 19 other conformations cover a range of RMSD from 0.9 to 17.1 Å (see Figure 3B). The closest conformation to the bound conformation, excluding itself, deviates by 0.9 Å and is from a ternary complex between von Willebrand factor, glycoprotein Ib alpha and botrocetin (PDB: 1U0N). Thus, BCLoopSearch appears able to return valuable collections of candidate loops. We recall however that only a rough pruning of the candidate loops is performed by BCLoopSearch and that further processing to score them should be considered.

### BCSpecificitySearch

**Figure 4.**
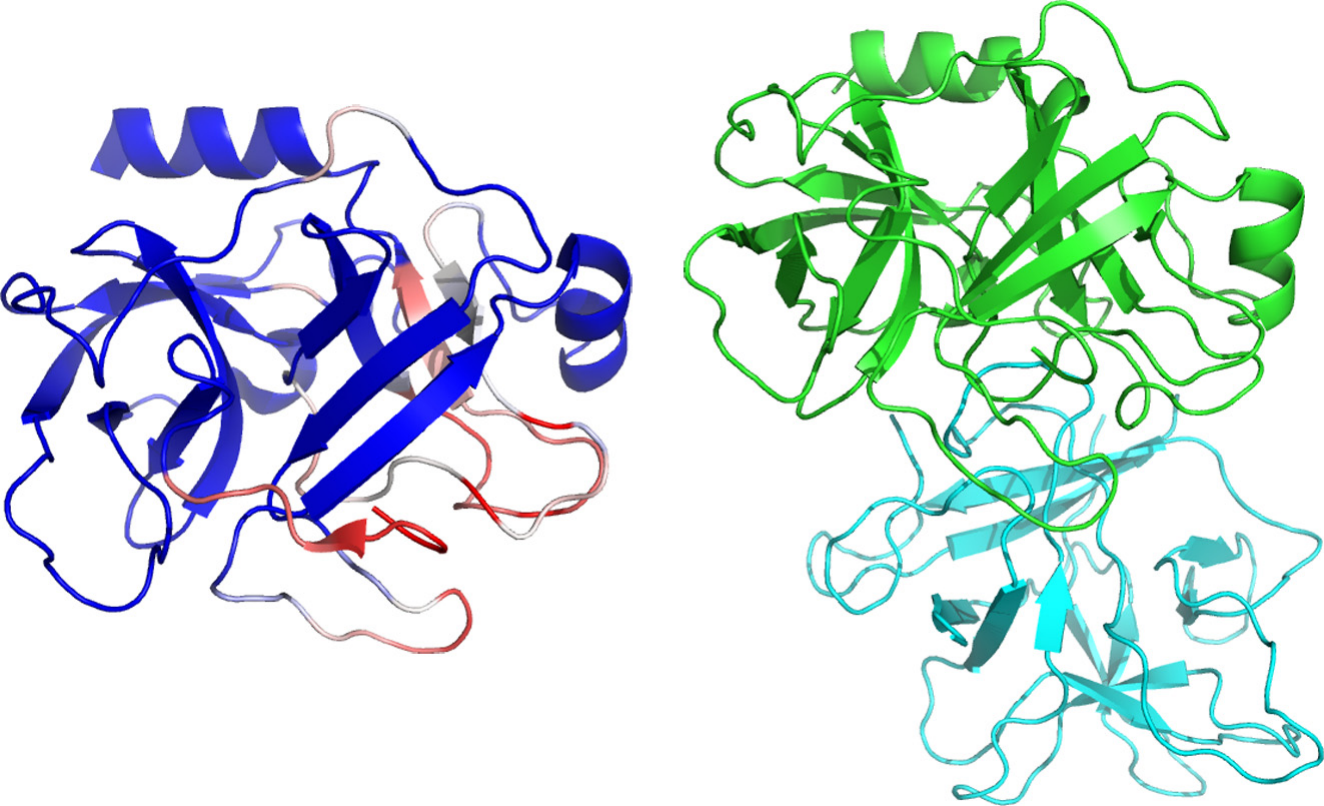
Left: Specific fragments of the porcine beta trypsin (PDB: 1QQU) as identified by BCSpecificitySearch. The red parts correspond to fragments specific of this conformation and the blue to non-specific ones. Right: Structure of the complex porcine pancreatic trypsin (green)/soybean trypsin inhibitor (cyan) (PDB: 1AVX).

The porcine beta trypsin (PDB: 1QQU) is associated with the SCOPe fold b.47. Using BCSpecificitySearch, it is possible to ask what fragments of the structure are specific of the fold, searching occurrences of fragments in the b class, but discarding the protein domains of the b.47 fold (5352 protein domains). We have used a fragment size of 9, against the corresponding SCOPe subset filtered at 90% sequence identity, BC-score and rigidity cut-off values of 0.95 and 1, respectively. Figure 4, left shows the sites associated with specificity scores greater than 0.995, i.e. associated with less than 0.5% of matches. It is striking that these sites define a patch on the structure. Interestingly, Figure 4, right shows that this patch corresponds to the patch in interaction with the soybean trypsin inhibitor (PDB: 1AVX).

## CONCLUSION

BCSearch services offer fast and versatile means to mine large collections of structures and extract information about local sequence–structure relationships. It is possible to search for fragments similar to a query, to search for fragments in a mirror conformation or to identify candidate fragments of fixed size matching the flanks of a gaped structure. BCSearch also provides an innovative means to analyze the specificity of local conformations in a complete protein structure by identifying sites associated with un-frequent conformations. Due to the properties of the BC-score, the parameters driving the search are few, and are independent of fragment size. Using the same framework, it is still possible to enlarge the panel of services. Particularly, all services presently perform ungaped search. As we have shown in one example, accepting a limited number of gaps could certainly help to extend the interest of BCSearch to motif identification from structure, a point for further development, however. It remains that BCSearch runs are typically on the order from few seconds up to few minutes only, depending on the collection of structures to mine, making it, we hope, suitable as a useful tool for biologists, to analyze, engineer or design proteins.
